# Using formative research with older adults to inform a community physical activity programme: Get Healthy, Get Active

**DOI:** 10.1017/S1463423618000373

**Published:** 2018-07-06

**Authors:** George J. Sanders, Brenda Roe, Zoe R. Knowles, Axel Kaehne, Stuart J. Fairclough

**Affiliations:** 1 Physical Activity and Health Research Group, Department of Sport and Physical Activity, Edge Hill University, Ormskirk, UK; 2 Faculty of Health & Social Care, Edge Hill University, Ormskirk, UK; 3 Personal Social Services Research Unit, University of Manchester, Manchester, UK; 4 The Research Institute for Sport and Exercise Sciences, Liverpool John Moores University, Liverpool, UK; 5 Department of Physical Education and Sports Sciences, University of Limerick, Ireland

**Keywords:** ageing, community groups, formative, physical activity, primary care

## Abstract

**Aim:**

The purpose of this formative study was to explore current knowledge and attitudes towards physical activity, as well as perceived barriers, facilitators and opportunities for physical activity participation among older adults living in the community. The findings have subsequently informed the design, delivery and recruitment strategies of a local community physical activity intervention programme which forms part of Sport England’s national *Get Healthy, Get Active* initiative.

**Background:**

There is a growing public health concern regarding the amount of time spent in sedentary and physical activity behaviours within the older adult population.

**Methods:**

Between March and June 2016, 34 participants took part in one of six focus groups as part of a descriptive formative study. A homogenous purposive sample of 28 community dwelling white, British older adults (six male), aged 65–90 years (M=78, SD=7 years) participated in one of five focus group sessions. An additional convenience pragmatic sub-sample of six participants (three male), aged 65–90 years (M=75, SD=4 years), recruited from an assisted living retirement home participated in a sixth focus group. Questions for focus groups were structured around the PRECEDE stage of the PRECEDE–PROCEDE model of health programme design, implementation and evaluation. Questions addressed knowledge, attitudes and beliefs towards physical activity, as well as views on barriers and opportunities for physical activity participation. All data were transcribed verbatim. Thematic analysis was then conducted with outcomes represented as pen profiles.

**Findings:**

Consistent views regarding both the potential physical and psychosocial benefits of physical activity were noted regardless of living status. The themes of, opportunities and awareness for physical activity participation, cost, transport, location and season/weather varied between participants living in an assisted living retirement home and community dwelling older adults. Further comparative research on the physical activity requirements of older adults living in assisted living versus community settings are warranted.

## Introduction

In the United Kingdom there are over 11 million older adults aged 65 years and over who make up 18% of the population (UK Office for National Statistics, 2017). Aligning with the United States and other developed countries (United Nations, 2015) this proportion is projected to increase to at least 24% by 2039 (UK Office for National Statistics, 2017). Although prolongation of life remains an important public health goal, of even greater significance is that extended life should involve preservation of the capacity to live independently, function well and quality of life (Rejeski *et al*., 2013). The purpose of this formative descriptive study was to explore current knowledge and attitudes towards physical activity (PA), as well as perceived barriers, facilitators and opportunities for PA participation among older adults living in the community. The findings were used to inform the design, delivery and recruitment strategies of an ongoing three-year community PA intervention project, *Get Healthy, Get Active* (GHGA), which forms part of Sport England’s national GHGA programme (Sport England, 2012).

## Background

Guidelines issued by the UK Chief Medical Officers and the US Surgeon Generals recommend that older adults (⩾65 years) engage in at least 150 min of moderate (or 75 min of vigorous) PA per week in bouts of at least 10 min, with muscle-strengthening and balance activities included on at least two of those days (Department of Health, 2011; Centers for Disease Control and Prevention (CDC), 2015). Despite the recognised evidence base for the benefits of regular PA (CDC, 2015; Reid and Foster, 2016; World Health Organization (WHO), 2017), objective summaries of PA levels among older adults show that only 15% of males and 10% of females within the United Kingdom, and 9.5% of males and 7% of females within the United States meet the recommended PA guidelines (Tucker *et al*., 2011; Jefferis *et al*., 2014). Given that current PA guidelines remain the same for both adults (18–64 years) and older adults (⩾65 years), such high levels of inactivity suggests that PA guidelines appear too demanding for the latter population (Booth and Hawley, 2015).

Accumulating evidence suggests that prolonged and continuous bouts of sedentary behaviours [SB; defined as waking behaviours in a sitting, reclining or lying posture with energy expenditure ⩽1.5 metabolic equivalents (Tremblay *et al*., 2017)] have similar physical (eg, premature mortality, chronic diseases and all-cause dementia risk) and psychosocial (eg, self-perceived quality of life, well-being and self-efficacy) risk factors to that of physical inactivity (Wilmot *et al*., 2012; Edwards and Loprinzi, 2016; Falck *et al*., 2016; Kim *et al*., 2016). In fact, SB is now an identifiable risk factor independent of other PA behaviours (Tremblay *et al*., 2017). Spending on average 80% of their time in a seated posture, and with 67% being sedentary for more than 8.5 h/day (Shaw *et al*., 2017), older adults are the most sedentary segment of society and seldom engage in moderate-to-vigorous PA (Chastin *et al*., 2017).

Several social (eg, social awkwardness and peer/family support), behavioural (eg, ageing stereotypes and lack of time), physical (eg, improved balance and flexibility) and environmental (eg, transport and neighbourhood safety) correlates of PA among older adults have been noted in recent formative (van Schijndel-Speet *et al*., 2014; Banerjee *et al*., 2015) and qualitative research (Franco *et al*., 2015; Devereux-Fitzgerald *et al*., 2016; Phoenix and Tulle, 2017). Such findings are a first step in enabling policymakers and health care professionals to implement effective PA interventions and promote active ageing (Franco *et al*., 2015). Given the potential benefits associated with PA outlined, such interventions have the potential to reduce, age-related morbidity and declines in activities of daily living, maintain muscle strength and mass, improve quality of life, and thus reduce the primary and total health care costs associated with SB and physical inactivity among this population (Bauman *et al*., 2016).

Prior research notes that interventions aimed at promoting PA participation should adopt an appropriate conceptual health promotion model to prioritise the key assets of the target group (Plotnikoff *et al*., 2014). The PRECEDE–PROCEED model of health programme design, implementation and evaluation (Green and Kreuter, 2005) provides the target population with a comprehensive and structured assessment of their own needs and barriers to a healthy lifestyle. The PRECEDE component of the model comprises of, predisposing, enabling and reinforcing factors has previously been used as a formative framework to guide PA intervention content and design (Mackintosh *et al*., 2011; Banerjee *et al*., 2015). This model has also been adopted as a method for the identification of perceived PA barriers and facilitators among older adults (Banerjee *et al*., 2015; Gagliardi *et al*., 2015) and other populations (Mackintosh *et al*., 2011; Emdadi *et al*., 2015; Susan *et al*., 2017).

The purpose of this formative study was to (i) explore current knowledge and attitudes towards PA, as well as the perceived barriers, facilitators and opportunities for PA participation among older adults living in the community who had agreed to take part in an ongoing PA programme; and (ii) use this data to inform the design, delivery and recruitment strategies of an ongoing community PA intervention programme, as well as international PA interventions among this population. Given the purpose and aims outlined, the Evidence Integration Triangle (Glasgow *et al*., 2012) was adopted as the overarching theoretical framework. Through the prompt identification of success and failures across individual-focussed and patient–provider interventions, as well as health systems and policy-level change initiatives, the framework allows for the exploration of the three main evidence-based components of intervention program/policy, implementation processes and measures of progress. Hence, this framework enabled a steep learning cycle through an initial 12-week pilot GHGA programme delivered by the Metropolitan Borough Council within the chosen local authority. Results and analysis from this pilot were fed back to Sport England as the funder, as well as deliverers and participants in order to assess, evaluate and promptly inform adapted future iterations of the GHGA programme.

## Methods

### Participants and procedures

A descriptive formative study was undertaken from March to June 2016. Participants were recruited from one local authority in North West England recognised as having the highest percentage of inactive older adults (80%) compared to the UK national average, and the highest national health costs associated with physical inactivity (Active People Survey, 2014; Sport England’s Local Profile Tool, 2015). The first author facilitated six, mixed-gender focus groups. Representative of the uptake of participants within the target GHGA initiative, a homogenous purposive sample of 28 community dwelling white, British older adults (five male) participated in five of the focus groups, with an additional convenience pragmatic sub-sample of six participants (three male) recruited from an assisted living retirement home, participating in the sixth focus group. In total, 34 older adults (eight male), aged 65–90 years (M=78, SD=7 years), participated across the six sessions. Four focus groups involved a group size of six to ten participants, and two involved three participants (mean focus group size of 6±5 participants). Previous focus groups in PA studies have been conducted effectively with as many as 12 (Moran *et al*., 2015), and as few as four (Schneider *et al*., 2016) participants. Focus groups took place in two church halls, an assisted living retirement home lounge, and a theatre. All locations were free from background noise, and participants could be overlooked but not overheard. The inclusion criterion set out by Sport England as funders of the GHGA programme were that participants must be 65 years of age or over, reside within one local authority in North West England, could provide written informed consent to participate.

GHGA is an ongoing three-year project which seeks to increase the number of inactive older adults participating in PA at least once a week for 30 min, via a 12-week PA intervention delivered by the Metropolitan Borough Council within the assigned local authority. Participants due to participate in GHGA received a covering letter, participant information sheet, and consent form. Prior to the commencement of the study, institutional ethical approval was received (#SPA-REC-2015-329) and written informed consent was obtained for all participants prior to participation. All focus groups utilised the PRECEDE stage of the PRECEDE–PROCEDE model (Green and Kreuter, 2005) within their design allowing for the exploration of predisposing, enabling and reinforcing correlates of PA participation. To maximise the interaction between participants, focus group questions were reviewed by the project team for appropriateness of question ordering and flow. Subsequent minor additions were made to questions on social isolation and PA advertisement. The semi-structured discussion guide included open ended questions structured to prompt discussion with equal chance for participants to contribute (Stewart and Shamdasani, 2014). Focus groups were led by a trained facilitator and with an observer/ note taker also present. Questions addressed knowledge, attitudes and beliefs towards PA as well as views on barriers and opportunities for PA participation. An example question from a section exploring barriers to PA was: ‘Can you tell me about what stops you from participating in physical activity?’ Questions therefore demonstrated aspects of face validity as they were transparent and relevant to both the topic and target population (French *et al*., 2015).

### Data coding and analysis

Focus groups lasted between 20 and 45 min (M=29, SD=12), were audio recorded, and later transcribed verbatim, resulting in 66 pages of raw transcription data with Arial font, size 12 and double-spaced. Verbatim transcripts were read and re-read to allow familiarisation of the data and then imported into the QSR NVivo 11 software package (QSR International Pty Ltd., Doncaster, Victoria, Australia, 2017).

Previous research within this population has adopted analytical procedures including thematic analysis (Van Dyck *et al*., 2017), content analysis (Middelweerd *et al*., 2014) and used specialist qualitative data analysis packages, such as NVivo (Warmoth *et al*., 2016). In supporting new methodologies and data representation within qualitative research (Orr and Phoenix, 2015), the current study followed the pen profiling protocol. The pen profile approach has been used in recent child PA research (Mackintosh *et al*., 2011; Boddy *et al*., 2012; Knowles *et al*., 2013; Noonan *et al*., 2016b) and presents findings from content analysis via a diagram of composite key emerging themes. In summary, data were initially analysed deductively via content analysis (Braun and Clarke, 2006), using the PRECEDE component of the PRECEDE–PROCEED model (Green and Kreuter, 2005) as a thematic framework which reflects the underlying study purpose. Inductive analysis then allowed for emerging themes to be created beyond the pre-defined categories. Data were then organised schematically to assist with interpretation of the themes (Aggio *et al*., 2016). As akin to more traditional qualitative research, verbatim quotations were subsequently used to expand the pen profiles, provide context and verify participant responses. Previous studies have demonstrated this method’s applicability in representing analysis outcomes within PA research (Mackintosh *et al*., 2011; Boddy *et al*., 2012; Knowles *et al*., 2013; Noonan *et al*., 2016a) making it accessible to researchers who have an affinity with both quantitative and qualitative backgrounds (Knowles *et al*., 2013; Noonan *et al*., 2016a). Recent findings suggest that the discrepancy between objective isolation and felt loneliness may be associated with undesirable health outcomes such as cognitive dysfunction.

Three pen profiles were developed to display themes within the data aligned to the PRECEDE component of the PRECEDE–PROCEED model (Green and Kreuter, 2005). Quotations were labelled by focus group number (Fn) and subsequent participant number (Pn) within that focus group. Characterising traits of this protocol include details of frequency counts and extracts of verbatim quotes to provide context to the themes. A minimum threshold for theme inclusion was based upon comparable participant numbers within previous research adopting a pen profiling approach (Boddy *et al*., 2012; Noonan *et al*., 2016a) and hence, was set as ⩾*n*=6, with n representing individual mentions per participant. However, multiple ‘mentions’ by the same participant were only counted once. Methodological rigour was demonstrated through a process of triangular consensus (Hawley- Hague *et al*., 2016) between the authors. This offered transparency, credibility and trustworthiness of the results, as the data were critically reviewed using a reverse tracking process from pen profiles to verbatim transcripts, providing alternative interpretations of the data (Smith and Caddick, 2012). The process was repeated through cross-verification and discussion until subsequent agreement on data themes in relation to verbatim extracts was reached (Aggio *et al*., 2016).

## Findings and discussion

### Predisposing correlates

**Figure 1:**
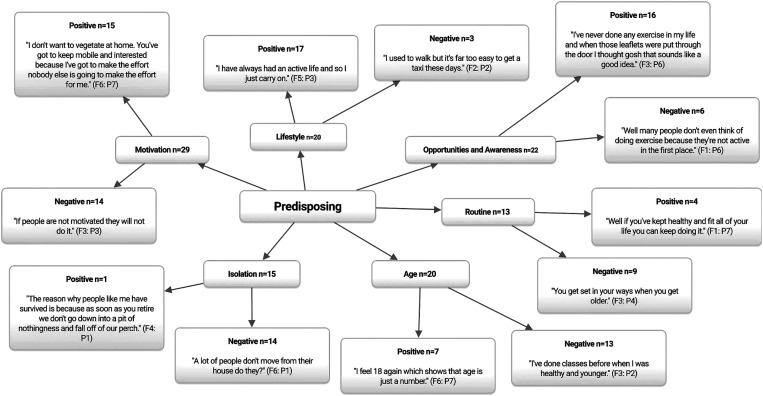
Predisposing correlates of physical activity participation among older adults. *n*=Individual mentions per person (multiple mentions not included); Fn=focus group number; Pn=participant number.

Figure 1 displays the predisposing correlates of PA participation. In agreement with previous research (Gray *et al*., 2015; Kosteli *et al*., 2016), the most highly cited theme of motivation (*n*=29) was perceived to be both a facilitator (*n*=15) and barrier (*n*=14) to PA participation throughout. Some participants were proactive in seeking out opportunities for PA.

*I’m a lung cancer survivor and I just ran a mile last month and I raised £550*.(Focus group (F) 1: Participant (P) 2)

Contrastingly, others expressed disinterest in PA altogether believing that they would not derive any health benefit.

*I’ve pushed these [PA] classes to lots and lots of friends and they still ignore it, they will not come to anything like this*. (F1: P3)

Participants also reported laziness or apathy to prevent participation.

*It’s [lack of PA] apathy, just apathy, people can’t be bothered*. (F4: P3)

The importance of pre-intervention intrinsic motivation (eg, participating for enjoyment) among older adults is key for both initial adoption and maintenance of PA participation (Gray *et al*., 2015). Hence, future interventions could promote intrinsic motivation for PA through the adoption of socio-emotional selectivity theory (Carstensen *et al*., 1999). Recent findings support this theory’s notion that motivation for PA is more effectively promoted when paired with positive messages about the benefits of PA rather than with negative messages about the risks of inactivity (Notthoff *et al*., 2016).

The theme of age (*n*=20) was identified as a key barrier (*n*=13) to PA participation throughout.

*They [older adults] get to a certain age and just give up*. (F1: P7)

Social norms and cultural misconceptions often influence not only the type of PA in which older adults engage, but whether they participate at all (Greaney *et al*., 2016). Moreover, participants noted that lifestyle (*n*=20) often affects individual views regarding ageing stereotypes, and therefore PA participation. Some participants felt that physically active older adults were more likely to be habituated to PA engagement over many years.

*Well if you’ve kept healthy, kept fit all your life, you can keep doing it*. (F1: P4)

Conversely, it was felt that inactive older adults were reluctant to start exercising.

*You see the ones who haven’t been doing it [PA] are not going to be able to start and do it now*. (F2: P1)

Previous research has also reported prior PA behaviours (eg, being sedentary or active) to be key correlates affecting older adults’ current PA participation levels (Franco *et al*., 2015). Additionally, ageing is associated with a decrease in the size of social networks and hence, older adults are at increased risks of isolation (Devereux-Fitzgerald *et al*., 2016; Greaney *et al*., 2016). Corroborating with prior research (Greaney *et al*., 2016), participants throughout perceived isolation (*n*=15) to be a key barrier (*n*=14) to PA participation.

*It’s so easy to get trapped inside and not go out. People sit in front of the television from the moment they wake up to when they go to bed*. (F6: P5)

Isolation is associated with decreased social and psychological well-being (Owen *et al*., 2010; Milligan *et al*., 2015) and increased SB among older adults (Nicholson, 2012). Certain targeted intervention strategies can reduce isolation by providing an opportunity for older adults from differing socio-economic areas to take part in PA within local community spaces (eg, parks, leisure centres and churches), that promote social networking by encouraging camaraderie, adaptability and productive engagement, without the pressure to perform (Milligan *et al*., 2015; Gardiner *et al*., 2016). Given that SB is an independent and modifiable behavioural target for interventions (Lewis *et al*., 2017), opportunities to replace SB with health-enhancing behaviours such as moderate-to-vigorous PA (Prince *et al*., 2014), light PA (McMahon *et al*., 2017; Phoenix and Tulle, 2017) and standing (Healy *et al*., 2015) should be promoted. However, none of the participants in the current study noted negative health effects of prolonged sitting, or the importance of breaks in sedentary time. Previous research has noted that older adults are not yet familiar with the concept of SB and hence, are not motivated to reduce such behaviours (Van Dyck *et al*., 2017). Hence, it is first crucial to increase knowledge about the negative health consequences of SB independent from PA among both older adults and other populations (Van Dyck *et al*., 2017).

Participants also emphasised the importance of having a wide range of choice and opportunities for PA (*n*=22), and in general their perceptions of community provision were positive (*n*=16).

*Yes it’s quite a good place [the local authority where the study took place]. There are a lot of different physical activity sessions to try*. (F2: P1)

However, in line with recent research (Baert *et al*., 2016; Träff *et al*., 2017), key barriers noted by the participants within the assisted living group included a lack of advertisement regarding PA opportunities, and few opportunities to take part in PA within the assisted living facility itself.

*It’s hard to know what is on if you don’t read the noticeboards and to be honest most of us have even stopped looking at that [noticeboard] because there is never anything on it*. (F3: P3)

Further research into the most effective advertisement strategies to engage older adults in assisted living facilities is warranted (Hildebrand and Neufeld, 2009). Regardless of living status, participants noted a strong preference not to engage with online and/or social media channels for advertising and awareness-raising.

*A lot of people our age don’t like that technology stuff at all. I would not know where to start.* (F5: P2)

These results suggest educational strategies outlining the potential benefits of technology in aiding PA participation are needed (Bird *et al*., 2015). This is especially salient given that recent research has shown technology-based interventions to have good adherence and provide a sustainable means of reducing SB and promoting PA participation among older adults (Garcia *et al*., 2016; Skjæret *et al*., 2016).

### Enabling correlates

**Figure 2:**
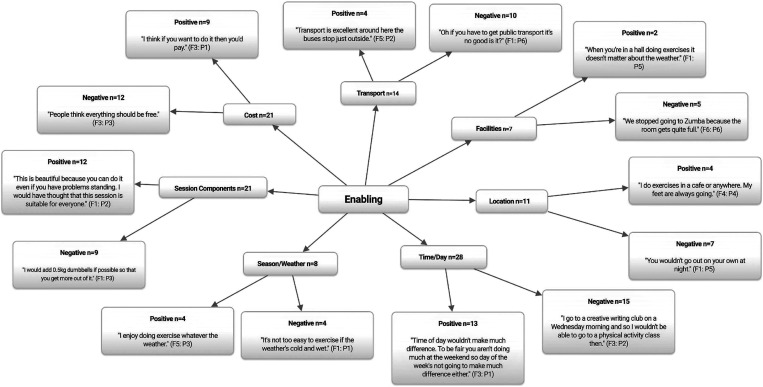
Enabling correlates of physical activity participation among older adults. *n*=Individual mentions per person (multiple mentions not included); Fn=focus group number; Pn=participant number.

Figure 2 displays the enabling correlates of PA participation. Consistent with previous research findings (Franco *et al*., 2015; Borodulin *et al*., 2016), cost (*n*=21) was perceived to be a key barrier (*n*=12) to PA participation exclusively among the community dwelling participants who were either unable, or unwilling to pay the perceived high costs associated with both attending and travelling to such programmes.

*Money is the big bug bear [barrier to PA participation] isn’t it*. (F2: P5)

Examples of competing programmes were also noted, with free and lower cost programmes taking precedence over the more expensive.

*We like it [a local chair-based PA programme] because it’s free*. (F4: P3)

Thus, to effectively increase PA participation within this population, health-promotion strategies should go further than merely educating and raising awareness about potential health benefits, and should also advocate for the provision of low-cost, and easy reachable PA opportunities regardless of financial status (Petrescu-Prahova *et al*., 2015; Borodulin *et al*., 2016). It is worth noting that for the participants recruited from the assisted living retirement home, any PA sessions delivered were included within the cost of the overall living fee, and hence lack of financial resources was rejected as a potential barrier for PA participation (Baert *et al*., 2016).

Participants’ views on the theme of location (*n*=11) centred on neighbourhood safety. Declining health and physical impairments associated with ageing increase the time spent in ones’ neighbourhood and thus, neighbourhood environmental factors such as, PA provision, proximity, traffic volume and overall neighbourhood safety are considered to be important correlates affecting older adults’ PA participation (Greaney *et al*., 2016). Perceived neighbourhood safety was identified as a barrier (*n*=7) to PA participation exclusively among the community dwelling older adults.

*You wouldn’t go out on your own at night around here.* (F1: P5)

Participants from the assisted living retirement home did not view neighbourhood safety to be either a barrier to or facilitator of PA. This neighbourhood environment was perhaps viewed as the norm and therefore they did not associate safety concerns so acutely (Moran *et al*., 2015). This association could have also affected results obtained for the theme time/day of the week as such participants did not recognise this to be a barrier to PA participation either.

*Time of day wouldn’t make much difference [to PA participation]. To be fair you aren’t doing much at the weekend so day of the week isn’t going to make much difference [to PA participation] either*. (F3: P1)

Conversely, community dwelling participants reported time/day of the week to be a barrier (*n*=15), with early morning or early evening sessions identified as reducing PA participation, especially during the winter months when daylight hours are more limited. These findings could have been further amplified by the neighbourhood safety concerns also identified by this group (Hoppmann *et al*., 2015; Prins and van Lenthe, 2015).

The theme of transportation (*n*=14) has been extensively reported to be both a barrier and facilitator to PA participation among older adults (Bouma *et al*., 2015; Haselwandter *et al*., 2015; Kosteli *et al*., 2016; Van Dyck *et al*., 2017). Within the current study transportation was identified as a barrier (*n*=10) restricting access to PA sessions regardless of living status.

*I would like to go to the baths [swimming pool] but it’s difficult to get there and back so I just don’t bother*. (F4: P5)

Transport is especially important for those lacking the ability to be more independently mobile as it allows individuals to bridge larger distances than they could by walking alone (Van Cauwenberg *et al*., 2016). Thus, lack of access to a car and inadequate availability, frequency and reliability of affordable public transport are all associated with decreased PA participation (Newitt *et al*., 2016). Additionally, being dependent upon others (eg, family, friends and peers) for transportation has been identified as a barrier to PA participation within this population (Baert *et al*., 2015). This was also noted in the current study.

*I think the worst thing is having to rely on somebody else to take you [to a PA session] as anything can happen in your own life let alone somebody else’s*. (F5: P2)

Prior research suggests the promotion of walking for transportation to PA sessions among physically independent older adults (Chudyk *et al*., 2017). However, given the neighbourhood safety concerns noted by participants, and the varying levels of functional ability among this population, further research examining access to PA sessions including walking facilities (eg, path and crossing quality), traffic safety and safety from crime is warranted (Van Cauwenberg *et al*., 2016).

### Reinforcing correlates

**Figure 3:**
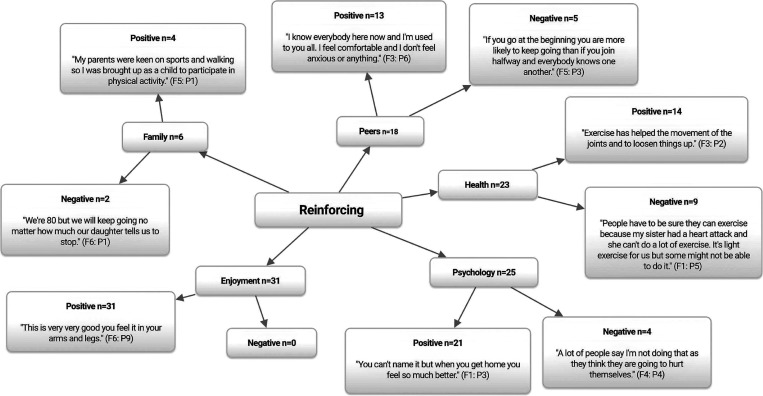
Reinforcing correlates of physical activity participation among older adults. *n*=Individual mentions per person (multiple mentions not included); Fn=focus group number; Pn=participant number.

Figure 3 displays the reinforcing correlates of PA participation. Peer support is associated with PA adherence in older adults (Brown *et al*., 2015), and was identified as a key theme (*n*=18) and subsequent facilitator (*n*=13) to PA participation in the current study.

*I’ve got to know everybody now and I’m used to you all. I feel more comfortable and I don’t feel anxious or anything*. (F3: P6)

Unsurprisingly, in light of the above several participants reported peers to be a barrier to PA participation (*n*=5) because of an unwillingness to attend other PA sessions due to anxieties about meeting new people.

*I wouldn’t like to go somewhere else as I wouldn’t like to walk in on a crowd of new people*. (F3: P6)

Although group-based activities offer older adults the chance to gain a sense of belonging, enjoyment and establish friendships, designing sustainable exit routes in order to retain the provision of group activities which continue to facilitate, build and retain social bonds post-intervention should be considered by PA programmers and policymakers (Wu *et al*., 2015).

In line with recent research (Devereux-Fitzgerald *et al*., 2016; Smith *et al*., 2017), family members were identified as being both barriers (*n*=2) and facilitators (*n*=4) to PA participation. Specifically, a barrier often reported is overprotectiveness, in which family members may not allow older adults to participate in PA out of concern for their safety or health (Greaney *et al*., 2016). Participants among the community dwelling groups also noted this.

*My sons in for a shock that we’re coming to this as he’s like, ‘no long walks, no boat rides’, he goes ‘you’re past it’.* (F6: P2)

Such results suggest a need to educate family members on the importance and benefits of PA among older adults. Educational resources such as the older adults PA guidelines infographics for the, United Kingdom (Reid and Foster, 2016), Canada (Canadian Society for Exercise Physiology, 2016), Australia (Australian Government Department of Health and Ageing, 2013), New Zealand (Ministry of Health, 2013) and the United States (CDC, 2008) are appropriate tools advocating for older adults to be active safely, and can be understood by family members plus health care providers. Furthermore, the adoption of local/national mass media messages may be a cost effective educational solution at a time when there is a growing ageing population (United Nations, 2015; UK Office for National Statistics, 2017). However, given the resistance to technology-based PA noted in the current study, further educational strategies promoting enjoyable, easy-to-use technology within a family environment are needed for community dwelling older adults (Bird *et al*., 2015). Participants within the assisted living group did not perceive family members to be either barriers or facilitators to PA participation and thus, further research is needed to identify approaches to involve family members as additional facilitators of PA participation within this group.

Participants viewed the theme of perceived health benefits (*n*=23) to be both a facilitator (*n*=14) and barrier (*n*=9) to PA participation regardless of living status. Participants were knowledgeable regarding the potential benefits of PA for their physical health.

*It [PA] loosens all your limbs up*. (F2: P2)

Participants also noted the potential benefits of PA for their psychological health.

*The wellbeing [from PA participation] makes you feel better*. (F1: P3)

Despite the irrefutable evidence demonstrating the benefits of PA among older adults (CDC, 2015; Reid and Foster, 2016; WHO, 2017), participants also noted health to be a potential barrier (*n*=14) to PA participation due to doubts about their capabilities, or fear of causing themselves harm, particularly if they were unfamiliar with it.

*People have to be sure they can come to PA sessions because my sister had a heart attack … and she can’t do a lot of these exercises*. (F1: P5)

To overcome such perceptions, educational strategies at a population level should focus on communicating the role of PA in gaining health benefits for all as well as how well-designed PA programmes can aid in the management of common comorbidities specific to this age group (Gillespie *et al*., 2012; Hamer *et al*., 2013).

Taken together with the findings of recent qualitative studies examining correlates of PA participation among older adults living in both assisted living (Baert *et al*., 2016; Träff *et al*., 2017) and community dwelling older adults (Fisher *et al*., 2017; Phoenix and Tulle, 2017), results from this formative research study have been used to inform the design, delivery and recruitment strategies of an ongoing community PA intervention project. Specifically, changes implemented to programme design have included the introduction of, increased intervention duration from 6 to 12-weeks, maintenance sessions post-initial 12-week intervention, tea and coffee after each session to promote social interaction, and a reduction of early morning and late afternoon sessions. Changes to programme delivery have included the introduction of, participant choice in session activities, videoing participants at week 1 and week 12 to show participants their progression, and signposting participants to other local PA programmes. Finally, changes implemented to recruitment strategies have included, improved relationships with general practitioners to enable them to refer participants onto the programme, leafleting in church halls and charity shops, and deliverers attending and subsequently advertising the programme at several Older Peoples’ Forums. Such methods could also be adopted throughout similar community PA programmes elsewhere in order to increase programme fidelity, representativeness and effectiveness.

## Strengths and limitations

Methodological strengths include the exploration of consensus and associated discussion through the focus groups and subsequent analysis process which allowed insight into the predisposing, enabling and reinforcing correlates of PA participation among older adults. Consistency of themes, data credibility, transferability, and dependability were achieved through the triangulation consensus of data between authors and methods. While this study reiterates important insights into the perceived barriers, facilitators and opportunities for PA participation among both community dwelling and assisted living older adults, value outside of this to the wider research community may be limited due to programme funding which only allowed for formative research strategies to recruit participants who had agreed to take part in an ongoing PA programme. Consequently, sampling bias is a potential issue as it could be assumed that a high proportion of the participants were already inclined to be and/or currently physically active given the positive predisposing comments with regard to motivation towards PA and current lifestyle choices (Costello *et al*., 2011). This is especially important given that motivators and barriers towards regular PA vary among currently active and inactive adults across the age range (Costello *et al*., 2011; Hoare *et al*., 2017). Considering that less than 10% of older adults (⩾ 65 years of age) meet the recommended PA guidelines (Jefferis *et al*., 2014), future research should seek to identify barriers and facilitators among larger sample sizes of currently inactive older adults living within both the community and assisted living facilities.

Additionally, a small convenience pragmatic sub-sample of participants from one assisted living facility were recruited and hence results cannot be considered representative. Furthermore, men tend to decrease participation in leisure-time PA as they get older; whereas this dose-response is not seen among women (Amagasa *et al*., 2017). Consequently, there is the possibility of gender bias given the higher number of female participants recruited. However, the sample size, participants’ ages and gender distribution are comparable to those reported in two recent studies examining barriers and facilitators to PA participation among older adults (Baert *et al*., 2015; Moran *et al*., 2015). Within these two studies the total number of participants was 15 (five male) and 40 (13 male), and the mean age of the respondents was 74 years, and 84 years, respectively. This compares to a total number of 34 participants (eight male) with a mean age of 78 years in the current study. Nevertheless, as well as exploring correlates of PA participation in relation to gender, functional status and age differences between the young–old (60–69 years), old–old (70–79 years) and oldest–old (80+ years) (Heo *et al*., 2017), future research should obtain additional participant characteristic data prior to the intervention including, participants’ current sedentary time and PA levels, history of PA, family history of PA, ethnicity, employment status and educational achievements as such have been shown to potentially affect the perceived barriers and facilitators to PA participation among older adults (Greaney *et al*., 2016; Keadle *et al*., 2016).

## Conclusions

Older adults acknowledged the benefits of PA, not only for health but also those relating to socialising, enjoyment, relaxation, and physical and psychological well-being. The themes of opportunities and awareness for PA participation, cost, transport, location and season/weather varied dependent upon living status. These findings suggest current living status to be a separate correlate of PA participation among older adults. This data can be used to further strengthen the design, delivery and recruitment strategies of both the target GHGA PA intervention programme and international PA intervention programmes among older adults. Future interventions should consider educational strategies to communicate the role of PA in gaining health benefits for all, reducing SB, and countering the negative implicit attitudes that may undermine PA within this population. Given the small sample of participants in the current study, further comparative research exploring the barriers and facilitators between assisted living and community dwelling, and active and inactive older adults on both national and international levels is warranted.
